# Uncovering the Holocene roots of contemporary disease-scapes: bringing archaeology into One Health

**DOI:** 10.1098/rspb.2023.0525

**Published:** 2023-12-06

**Authors:** Kristen M. Rayfield, Alexis M. Mychajliw, Robin R. Singleton, Sabrina B. Sholts, Courtney A. Hofman

**Affiliations:** ^1^ Department of Ecology and Evolution, Stony Brook University, Stony Brook, NY, USA; ^2^ Laboratories of Molecular Anthropology & Microbiome Research, University of Oklahoma, Norman, OK, USA; ^3^ Department of Anthropology, University of Oklahoma, Norman, OK 73019-0390, USA; ^4^ Department of Anthropology, National Museum of Natural History, Smithsonian Institution, Washington, DC, USA; ^5^ Department of Biology & Program in Environmental Studies, Middlebury College, Middlebury, VT 05753-6203, USA

**Keywords:** niche construction theory, zoonotic diseases, disease ecology

## Abstract

The accelerating pace of emerging zoonotic diseases in the twenty-first century has motivated cross-disciplinary collaboration on One Health approaches, combining microbiology, veterinary and environmental sciences, and epidemiology for outbreak prevention and mitigation. Such outbreaks are often caused by spillovers attributed to human activities that encroach on wildlife habitats and ecosystems, such as land use change, industrialized food production, urbanization and animal trade. While the origin of anthropogenic effects on animal ecology and biogeography can be traced to the Late Pleistocene, the archaeological record—a long-term archive of human–animal–environmental interactions—has largely been untapped in these One Health approaches, thus limiting our understanding of these dynamics over time. In this review, we examine how humans, as niche constructors, have facilitated new host species and ‘disease-scapes’ from the Late Pleistocene to the Anthropocene, by viewing zooarchaeological, bioarchaeological and palaeoecological data with a One Health perspective. We also highlight how new biomolecular tools and advances in the ‘-omics’ can be holistically coupled with archaeological and palaeoecological reconstructions in the service of studying zoonotic disease emergence and re-emergence.

## 1. Introduction

The profound environmental alterations that characterize the Anthropocene—our human-driven epoch of intensive agriculture, deforestation, pollution and extractivism—have introduced new variables into zoonotic pathogen selection [1,2]. Consequently, novel human pathogens have emerged from wildlife over the last century with increasing frequency, including HIV, pandemic strains of H1N1 influenza A virus, ebolaviruses and highly human pathogenic coronaviruses. Today zoonotic pathogens account for at least 60% of emerging infectious diseases in humans, and significant efforts are directed at predicting and preventing new threats based on the phylogeny, ecology, environmental conditions and human interactions of different host species and populations [3].

The One Health approach centres the inextricable interconnectedness of human, animal and environmental health and thereby guides multi-disciplinary investigations of many different factors in disease emergence [4]. While this approach has been successful in addressing syndemics—where multiple diseases cluster together and influence public health—the temporal dimension of zoonotic disease emergence is often underappreciated, if not entirely overlooked [5,6]. Archaeologists can help address this problem by documenting the deeper history of humans in shaping diseases. However, archaeological methods, tools and data have not fully been leveraged in One Health approaches, with a few notable exceptions such as the One Health Archaeology Research Group at the University of Edinburgh and a handful of initiatives and researchers elsewhere [7–11].

The archaeological record is an archive of the long-term human–animal–environment interactions that provides a temporal context for understanding disease dynamics in a particular place, culture or ecosystem. Anthropogenically created disease landscapes, or ‘disease-scapes’ [12], are the result of the types of animals, plants and microbes that are culturally and behaviourally selected within a constructed niche—a process known in archaeology as niche construction theory. Zoonotic transmission is often a complex cascade of events that requires the alignment of ecological, epidemiological and behavioural determinants that can increase pathogen pressure, host exposure and host receptivity to infection [13]. These disease dynamics are not novel but rather build upon successive rounds of anthropogenic disruptions over time. Indeed, major shifts in human behaviour and their environmental impacts have deep roots in the current Quaternary period (approx. 2.58 Ma to present) [14–16], long before the Great Acceleration of 1950 CE [17]. Many of the drivers of disease emergence we see today, including deforestation, species translocation and urbanization [18,19], are likewise ancient [20,21]. The present is therefore a palimpsest of overwritten human interactions with landscapes and species.

Here, we outline a technical toolset that demonstrates how One Health research can be extended by uniting zooarchaeological, bioarchaeological and palaeoecological data from the Late Pleistocene (approx. 126 000 to 11 700 years ago) to the Anthropocene (which has various proposed starting dates) [15,17]. Such temporal dimensions and diverse datasets can elucidate early associations in human-driven disease dynamics as models for future research. Importantly, we also address the relevance of the archaeological lens in highlighting health disparities, as past pandemics have disproportionately affected marginalized communities and created inequities that persist into the present [22,23]. With a focus on these issues, we provide steps forward for interdisciplinary research and highlight ethical considerations for collaborative and inclusive research.

## 2. Epidemiological transitions as manifestations of human niche construction through time

Niche construction theory (NCT) provides a co-evolutionary framework in which organisms do not solely adapt to their environment, but rather reconstruct the environments around them, which in turn creates or influences other natural selective pressures [24,25]. It recognizes that the selective pressures created during niche construction have long-lasting effects on multiple taxa and result in greater evolutionary and ecological consequences [26,27]. Consequently, not only are selected genes passed from generation to generation, but so too is an altered ecological inheritance [24,26]. Organisms create repetitive niche constructions and the environment is then re-imposed on future generations, becoming a force of selection [26].

**Figure 1.**
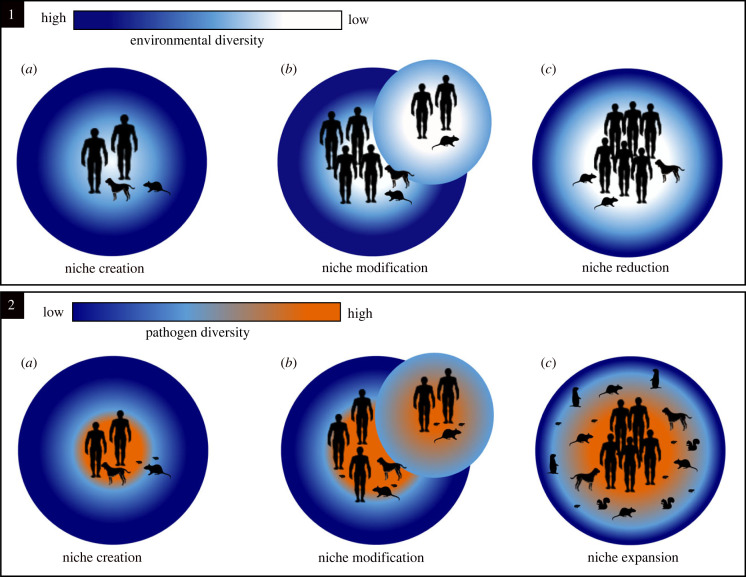
Human niche construction of disease-scapes. Anthropogenic impacts on disease dynamics can be explored through niche creation, niche modification and niche reduction (1). While ecological niches are further reduced, pathogen niches expand (2). During niche creation a subset of an area is altered (1a); consequently, introducing naive host populations to new pathogens (2a). Through niche modification, specific plants and animal species are selected, resulting from either domestication, translocation, or extinction events (1b), thus resulting in an influx in zoonotic spillovers (2b). Within niche reduction, humans continue to limit species richness within the constructed niche (1c), yet pathogen biodiversity expands as new reservoir hosts are created and diseases become endemic (2c).

Humans have greatly influenced their own evolution by altering environments for their benefit, which in turn has created and influenced other selective pressures [16,28]. Archaeology recognizes the impacts of these alterations within disease ecology through an epidemiological transition framework. We can therefore consider epidemiological transitions as manifestations of human niche construction through time, where humans have driven disease dynamics and pathogen selection within their shared environment through niche creation, niche modification and niche reduction (figure 1). Three epidemiological transitions are recognized with parallel landscape alterations and human demographic transitions: (1) a rise in zoonotic diseases as humans adopted agriculture and transitioned to more sedentary lifestyles beginning more than 10 000 years ago, (2) a shift from acute infectious diseases to chronic diseases with Western industrialization and colonialism over the last several centuries, and (3) an increase in (re-)emerging infectious diseases due in part to antibiotic resistance and global travel in recent decades [29,30]. As the Third Epidemiological Transition continues to unfold, it is critical to recognize that these transitions are a consequence of continued alterations to modified disease-scapes.

### (a) First Epidemiological Transition

The transition from the Late Pleistocene to the Holocene (approx. 11 700 years ago) is associated with major climatic shifts, the extinction of the majority of the world's mammalian megafauna, and human population size increase. Through complex and variable processes that continue to be studied, new developments in lithic technologies and subsistence strategies become evident in the archaeological record for this time period, including a transition from foraging to increased sedentism and localized food production [28]. While agriculture developed independently and asynchronously in different regions (with the earliest evidence in the Levant approx. 12 000 years ago and the most recent in North America approx. 5000 years ago [29]), this transition is acknowledged by the onset of selective breeding of certain traits in plants and animals in addition to the rise in monocrop strategies and reduction in the diversity of food items consumed [28].

These anthropogenically altered landscapes (also known as anthromes) introduced humans to both vector and non-vector parasites through irrigation systems and faeces used as fertilizers [29]. Parasitic infections, such as blood-borne *Plasmodium* parasites that cause malaria, thus became more predominant in human–animal–environment interactions [31]. Burning, clearing, terracing and irrigation resulted in habitat fragmentation while also redefining human, domestic animal and wildlife relationships. Landscape alterations enhanced opportunities for domesticated animals such as cattle, goats, sheep, pigs and poultry to become reservoirs and intermediate hosts for zoonotic diseases such as influenza, tuberculosis and brucellosis [29,32]. Growing human and animal populations in crowded and confined spaces became conducive to the spread of crowd diseases and the evolution of more virulent human pathogens, such as those that cause measles and smallpox [29].

Such changes sparked the First Epidemiological Transition, which has been defined as an influx of infectious diseases observed within the bioarchaeological record. For instance, compared with Palaeolithic foragers, the skeletal remains of early agriculturalists have provided evidence of increased nutritional deficiencies, oral pathologies, chronic infections, co-morbidities and childhood mortality rates [29]. The adoption of agriculture thus changed disease ecology as humans created new routes and sources of infection, where human waste, food stores and livestock became prominent within densely populated communities.

### (b) Second Epidemiological Transition

From the fifteenth to twentieth centuries CE, extensive settler colonialism and industrialization required more elaborate built environments to support large, densely packed populations, which experienced growing social and health disparities [33]. Many infectious diseases (e.g. smallpox, typhoid, typhus and tuberculosis) became established with reoccurring outbreaks due to the harsh working environments, crowded living conditions and the geographical spread of pathogens through Euro-colonial expansion [33]. Simultaneously, medical interventions, improved nutrition and public health measures led to increased life expectancy and population size around the world, with demographic changes towards greater proportions of older individuals with chronic illnesses. This Second Epidemiological Transition is characterized by a shift from these periodic infectious outbreaks to a prevalence of chronic, non-degenerative, non-infectious diseases such as cancer, diabetes, obesity, and diseases related to environmental pollution [33,34].

### (c) Third Epidemiological Transition

The Third Epidemiological Transition is associated with the emergence of novel and familiar diseases in different geographical areas as well as new threats of antibiotic-resistant pathogens as a result of rapid globalization [30,35]. This epidemiological transition is most often linked to changes in disease ecology and major anthropogenic disruptions, such as human-driven climate change and consequent changes in host and vector distributions, industrialized food production and related pathways for food-borne and livestock-mediated diseases, as well as global travel and trade networks that can rapidly spread zoonotic pathogens to and among people everywhere. In addition, increasingly sanitized environments to mitigate outbreaks, as well as increasingly intensive farming methods to increase the size and quantity of livestock, have led to widespread overuse and misuse of antibiotics and thus diminished microbial diversity within human and human-associated populations. The loss of microbial diversity within the gut microbiome is thought to be associated with the rise in allergies and autoimmune diseases, as early childhood exposure to microbes are essential for training and strengthening the immune system (also known as the hygiene hypothesis) [36,37].

### (d) Overlapping epidemiological transitions

Epidemiological transitions can overlap at varying geographical scales [35]; for instance, the impacts of land appropriation and colonial expansion brought new diseases into immunologically vulnerable populations. Such translocations resulted in devastating consequences, such as the decimation of Indigenous people in the Americas from measles, smallpox and other diseases introduced via European colonization beginning in the fifteenth century [38,39] (box 1). New global supply chain demands, mass mining and over-reliance on monoculture have led to global landscape changes that are still felt today with modified trophic interactions (e.g. the biodiversity loss of predators and consequent propagation of pathogen-carrying reservoirs such as mice, rats, bats) [40], and the rapid progression of outbreaks to epidemics and pandemics due to globalization [41]. These overlapping epidemiological transitions and disease dynamics (including exposure, transmission and host switching) frame the importance of a temporal dimension to One Health, but they can only be teased apart with the inclusion of zooarchaeological, bioarchaeological and palaeoecological datasets.

Box 1. **The origin and spread of morbilliviruses**

**Figure 2.**
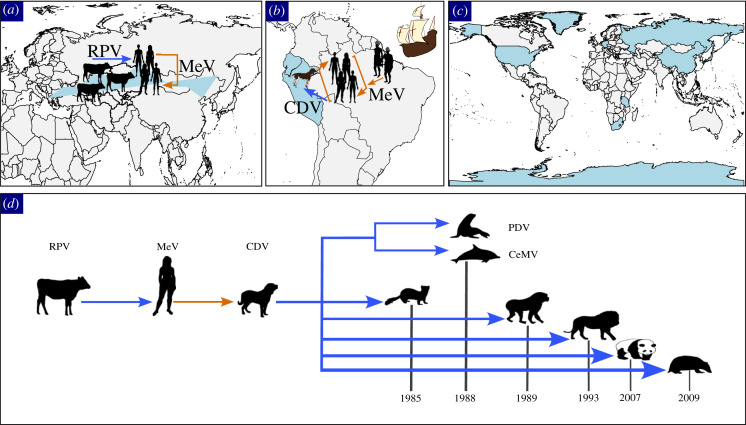
Origin and spread of morbilliviruses. Arrows represent transmission direction, with blue for animals and orange for humans. (*a*) Transmission of rinderpest virus (RPV) to humans and the origin of measles virus (MeV). (*b*) Transmission of MeV into the Americas and the first documentation of canine distemper virus (CDV). (*c*) Current reported countries with CDV. (*d*) Spread of morbilliviruses into recent alternative host species.

Humans have driven disease dynamics and pathogen selection with the origin and spread of crowd diseases like morbilliviruses, which cause rinderpest (RPV), measles (MeV) and canine distemper (CDV) (figure 2). RPV is a ruminant morbillivirus that was eradicated in 2011 [42]. The origin of MeV has been associated with the early transmission of RPV from domesticated cattle in the Eurasia Steppe during the sixth century BCE [39,43]. The spread of MeV from Europe to the Americas caused massive mortality among Indigenous people in the sixteenth century CE. This MeV outbreak may have spilled over into local dogs, as the first recorded case of CDV is noted in Ecuador during this time [39]. Since its transmission into domestic canines, the transmission of CDV into alternative hosts has rapidly and globally expanded. Outbreaks have been reported within marine mammals, including Phocid (seal) distemper virus (PDV) and Cetacean morbillivirus (CeMV), as well as in ferrets, tigers and lions, pandas, badgers and non-human primates.

## 3. Tools and datasets to reconstruct past disease-scapes

**Figure 3.**
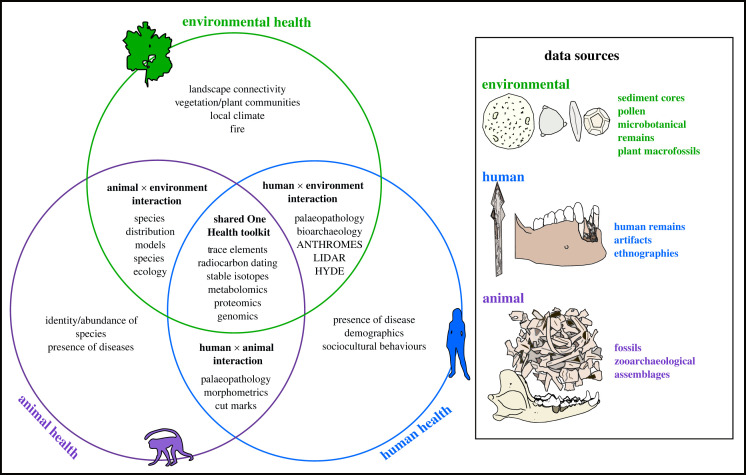
Tools and datasets to reconstruct past disease-scapes. The Venn diagram presents tools that can be implemented into a One Health framework to reconstruct past disease-scapes. The box contains data sources for each component—human, animal, environment—that can be studied.

Examination of the archaeological record allows researchers to trace niche creation, modification and reduction through time. Below, we offer a toolkit to explore how past diseases and humans could have reciprocally shaped each other (figure 3). We then advocate for how archaeological tools and datasets can contribute to documenting changes in environmental, animal and human health in the past.

### (a) Reconstructing past animal populations and health

Zooarchaeology is the study of animal remains (e.g. bones, feathers, eggshells, hides and shells) within archaeological contexts, such as harvest, consumption, domestication, translocation or extinction events [44]. This research permits us to reconstruct past species richness and abundance, which can be used to evaluate hypotheses exploring the underpinnings of human interactions with different animal species (i.e. food, dairying, ritual, clothing, shelter, tools, cultural material). These findings provide insight into types of spillover opportunities (e.g. subsistence strategies and dietary patterns) that may have been present. Zooarchaeologists most often employ osteological approaches for species and element identification, which help to determine minimum number of individuals (MNI) within the assemblage, body size, sex ratios, age at death, skeletal modifications and pathologies, and signs of domestication [45]. The majority of zooarchaeological faunal assemblages consist of fragmented skeletal remains, which can create challenges to determine species identification. Both cultural (e.g. butchering, modelling for tools) and taphonomic (e.g. diagenetic processes and bioturbation) filters can make taxa identification even more challenging [46]. However, taxonomic resolution may be achieved by analysing the differences in mass of the collagen peptide, which forms a unique fingerprint for different taxa [46]. Zooarchaeology by mass spectrometry (ZooMS) has been useful in reconstructing past ecologies by looking at species that are otherwise infrequently observed in archaeological faunal assemblages using osteological approaches [47]. It has provided taxonomic resolution for animal remains that have been culturally modified (tools, jewellery) [48,49], as well as for animals that are difficult to distinguish through skeletal remains alone (sheep and goat) [46,50]. Yet, the application of ZooMS is limited to taxa where there is enough divergence within the collagen protein. Therefore, canid species (dog, wolf and coyote), bovids (cattle and bison) or equids (horse and donkey) would be difficult to identify at the species level using ZooMS alone. Alternative peptide biomarkers may resolve some of these taxonomic resolution issues in equid or bovid species [51].

Combined zooarchaeological and palaeogenomic approaches have been used to study infectious disease within animal remains [52–54]. Early veterinary reports and more recent palaeopathology studies of animal diseases include canine distemper, rabies, cowpox, tuberculosis and plague, to name a few [39,55]. Some challenges within the discipline include disease identification due to skeletal preservation and taphonomic changes which can impact pathogen palaeogenomic research as well as the varying physiology of the pathology among different species (i.e. the degree of pathogenicity) [56]. Here, the integrative research among veterinarians and zooarchaeologists would greatly benefit zoonotic disease research. Animal health experts can provide uniform disease recording methods among different species while zooarchaeologists can provide context for early human–animal interactions [56]. Reconstructing microbial genomes and pathological manifestations in the skeletal remains of wild and domestic animals can elucidate disease origins, alternative enzootic transmission routes, ecological changes and human–animal interactions that may have facilitated cross-species transmission in the past.

### (b) Reconstructing past human populations and health

The bioarchaeological record—human skeletal remains within archaeological contexts—can reconstruct past human health and behaviours that may have impacted past and future disease transmission events. Bioarcheological methods have provided insights into human origins [57], human migration [58], human behaviours including funerary and ritual practices [59], identity and gender roles [60], diet [61], demography [59] and the impacts of these behaviours on past human health [62]. Many findings have resulted from osteological analyses, yet the incorporation of biomolecular techniques is providing novel insights into early disease dynamics.

Within the subfield of palaeopathology, the study of pathological conditions in ancient remains, most techniques have relied on identifying skeletal alterations in response to environmental factors during life. These bony lesions can result from trauma (e.g. fractures and breaks) and nutritional stress, which can lead to metabolic diseases such as rickets, scurvy, osteoporosis, osteomalacia and fluorosis [61]. Bioarchaeological research on nutritional stress has focused on cribra orbitalia, porotic hyperostosis, stature and oral pathologies as indicators of poor health and increased susceptibility in past populations [61,62]. Although most people died of infectious diseases prior to the development of germ theory and modern medical interventions, palaeopathology has focused mostly on certain bacterial, fungal and parasitic infections with specific indicators in bone (e.g. tuberculosis, leprosy, brucellosis and treponemal diseases—particularly syphilis). This focus is partly due to the lack of bony responses to acute infections that typically occur with viral pathogens. Researchers are thus often limited in their abilities to study viral infections using skeletal remains alone, with the exception of poliomyelitis and variola osteomyelitis [63]. Radiological imaging can help to some extent by visualizing features that are not accessible through anthroposcopic techniques.

### (c) Reconstructing past environments

Sediment cores of both anthropogenic and natural contexts hold great promise for the reconstruction of disease-scapes, as they represent incremental snapshots of environmental changes and can therefore be used to evaluate the environments in which human–pathogen relationships evolved. These stratigraphically controlled samples of sediments can be taken from lakes, oceans and terrestrial environments to reconstruct past vegetation, climatic conditions and fire regimes. Charcoal particles provide some of the earliest evidence of landscape alteration in the form of controlled burning and deforestation up to 45 000 years ago in Southeast Asia, Australia and Papua New Guinea [64] and among Pre-Columbian settlements in the Amazon [64,65]. Such analyses have highlighted the longevity of human manipulation of tropical forest ecologies, especially when combined with macrobotanical remains (seeds, roots, woods and fruits) and microbotanical remains (phytoliths, pollen, spores and starch grains) [44]. Botanical remains can also provide evidence for plant species translocations through time (native, alien and invasive species). The botanical remains found on tools or ceramics can reveal the types of plants that people ate and used (such as for ritual and medicinal purposes) in the past. Together, these datasets can reconstruct how plant communities changed over time, laying a foundation for palaeoecological reconstructions.

Estimating the human impact on environments via proxies of population size can also be achieved through a diverse combination of methodologies, ranging from laser imaging detection and ranging (LiDAR) for archaeological site identification and size estimation, estimation of faecal accumulation from stanols [66], and summed probability distributions (SPDs) of radiocarbon dated materials from archaeological sites [67,68]. With many open-source databases, archaeological data can now be modelled to explore regional and global population fluctuations and responses to environmental and climatic changes. These include palaeoecology databases like Neotoma [69] and the International Paleofire Network [70], radiocarbon databases such as p3k14c [71], and environmental models such as History Database of the Global Environment (HYDE 3.2) [72] and ANTHROMES [73]. For example, palaeoenvironmental reconstructions using ANTHROMES data illustrated that more than 95% of temperate and 90% of tropical woodlands were inhabited or cultivated as far back as 12 000 years ago [73]. Combining these palaeoenvironmental datasets with disease records can point to possibilities for disease emergence in ancient times. For instance, periods of drought and climatic shift, which can be identified through dendrochronology and sediment cores, are usually followed by famines and high mortality rates, as observed during the Late Antique Little Ice Age during the sixth century CE [74]. The presence of controlled fire and deforestation for agricultural and irrigation purposes signifies large population sizes, pooling water and the propagation of vector-borne diseases, as observed with malaria [29]. In addition, current deforestation changes bat roosting patterns into more favourable habitats for various bat species, thus potentially allowing a greater likelihood for bat-borne viruses to enter human populations [75]. Therefore, palaeoecological datasets and environmental reconstructions can elucidate some of the early baselines for current disease-scapes.

### (d) Integrative tools to reconstruct past human–animal–environment interactions

The incorporation of geochemistry and new ‘-omic’ methodologies has advanced our understanding of past human–animal–environment interactions within the same geographical area. While there is a plethora of research on how each of these tools have been implemented in different scientific disciplines, we focus on geochemical and ‘-omic’ tools that can be incorporated in reconstructing past disease-scapes by knitting together data across humans, animals and their shared environments.

#### (i) Geochemistry

Geochemical techniques including stable isotopes and trace element analysis can be used to reconstruct past diets, life histories, migration, climates and husbandry strategies. Stable isotope analyses of organic remains provide quantitative perspectives of the life histories and resources consumed [44]. Bulk stable isotopes of carbon and nitrogen are most frequently used to analyse dietary patterns and trophic levels, though new studies are increasingly using compound-specific stable isotopes to address changes in trophic position within amino acids [76]. Strontium, oxygen and hydrogen isotopes have reconstructed past migration, trade and diet within humans and animals, as they reflect the geochemistry of local geology and hydrology [77]. Trace elements, such as cadmium, lead, manganese, mercury and zinc, can help identify risks to human health, given that they are needed for metabolic processes, but their overabundance in the environment can be harmful, as observed with lead pollution from Roman mining and metallurgy (100 BCE–400 CE) [78,79].

#### (ii) Omics

New techniques in the ‘omics’ have opened doors to untangle past human–animal–environment interactions with relevancy for uncovering past epidemiological events [7,80]. This revolution has predominately been led by archaeogenomics made possible by ancient DNA (aDNA) from bone, dental calculus, seeds and palaeofaeces from animals, plants and humans. Now environmental DNA (eDNA, also known as sedaDNA) methods can reconstruct past ecologies where zooarchaeological and bioarchaeological data may be absent [81]. With more standardized methods, targeted enrichment techniques and high-throughput sequencing, ancient genome analysis has allowed researchers to explore human and animal phylogeographic patterns [82] as well as the reconstruction of ancient pathogen genomes. These methods have been applied to *Yersinia pestis* [83], *Mycobacterium tuberculosis* [80], human herpes simplex virus 1 (HSV-1) [84], the pandemic influenza strain of 1918 [85], *Salmonella enterica* [86], *Klebsiella pneumoniae* [87] and more. Many of these pathogens can infect multiple species that have long and close relationships with humans (i.e. *Y. pestis* in rodents, *M. tuberculosis* and *Brucella* in cows) and have been recovered from both archaeological and zooarchaeological assemblages [7,32,80].

Where aDNA is limited due to preservation, other omic techniques such as palaeoproteomics and metabolomics are starting to fill in the gaps, especially since proteins may preserve better than DNA [88]. Both targeted and metaproteomic techniques have been applied to reconstructing our own evolutionary history as well as determining how past animal by-products have been used in tool manufacturing, art and dairying [88–90]. The application of metaproteomics allows researchers to investigate ancient diseases through host–pathogen interactions as well as explorations of the host immune response [91]. Metabolomics has proven value in highlighting pathogen biomarkers [92]. Within the archaeological record, metabolomics has mainly been used for the analysis of organic residues found within ceramics. Yet it can also be applied to recover gut microbiota from palaeofaeces [93], which may be beneficial in studying shifts in human and animal microbiomes and any association to disease susceptibility.

Palaeo ‘omic’ studies are proving their value for current health concerns [94]. For instance, palaeogenomic studies on host–pathogen relationships have provided novel insight in pathogen selective pressures on human genome diversity and present-day inflammatory disease [95,96]. With the help of artificial intelligence, palaeogenomic datasets have yielded novel peptides with antibiotic properties, opening new pathways for drug development [97]. Further integration of these host and pathogen palaeo ‘omic’ studies has great potential in addressing patterns of increased virulence and disease susceptibility for current and future epidemiological concerns.

### (e) Evaluating synergies: how did human–animal–environment interactions contribute to zoonotic spread? A focus on rats of the genus *Rattus*

Humans have been translocating species for at least 20 000 years [16,98–100]. Four species of rodents—the black rat (*Rattus rattus*), the Pacific rat (*Rattus exulans*), the house mouse (*Mus musculus*) and the brown rat (*Rattus norvegicus*)—are significantly more abundant in anthropogenically modified niches where they exist as commensal organisms. In archaeological contexts, their remains are often found in food storage pits and linked to agricultural transitions and food surplus [99,101]. These species of rodents have attained a global distribution due to human migration and have therefore been successful in inhabiting areas outside their natural bounds with human assistance [101]. They have been used as a proxy to study human migration [102], but can also serve as a proxy for associated vector-borne diseases [98,102]. Rodents are characterized as hyper-reservoirs given that they carry many zoonotic disease agents that can infect humans, including those that cause hantavirus diseases, viral haemorrhagic fevers, leptospirosis plague and others [103]. Through human-facilitated translocation, they have contributed to extinction events and the establishment of new disease reservoirs. Examples include the North American introduction of *Y. pestis* via infected rats and their fleas in the early twentieth century, when ground squirrels and prairie dogs became plague reservoirs that persist in the western USA to this day [104] (box 2), as well as the extinction of Christmas Island rat (*Rattus macleari)* following the transmission of a pathogenic trypanosome carried by the recently introduced black rats in 1900 CE [105].

Box 2. **What can we learn from past pandemics: plague pandemics**

Many of the techniques described in §3 have helped to uncover the long-lasting impact and ecological inheritance of the first and second plague pandemics. Research in pathogen DNA, rat ecology, environmental changes and vector competence are unravelling the role of host susceptibility and vector dynamics within a shared environment [106]. Through the analysis of documented plague burial sites and extraction of aDNA from victims of the Black Death (1347–1351 BCE) at the onset of the second plague pandemic, Bos *et al*. [83] reconstructed the first draft genome of *Y. pestis*. This strain and the more recent *Y. pestis* strains appear to have been more virulent and transmissible compared with ancestral strains that circulated in Eurasia more than 5,000 years ago, possibly reflecting changes in disease ecology [107]. Reconstructed partial *Y. pestis* genomes recovered from rat skeletal remains add new information to the evolution of *Y. pestis* strains and the role of rats as a reservoir [108]. Palaeogenomic analysis of black rat populations reflect human migration and trade networks established during the Roman Empire [109]. The spatial distribution of rat populations may highlight regions where plague outbreaks (including the Justinian Plague of the first plague pandemic in 541–549 CE) occurred due to the congregation of both human and rat populations and the cross-regional transportation routes between them. Palynological records also support spatial heterogeneity in the impact of *Y. pestis* outbreaks [110]. The abundance of crop pollen in sediment cores can be used as a proxy for population size—the greater agricultural turnover, the larger the population—thus suggesting the Black Death had differential impacts across populations and communities. Recent investigations into the human genome using aDNA has also suggested lasting effects of susceptibility to the plague with implications for autoimmune disease today [96]. This research has continued relevance, along with the ecological changes that created long-lasting social and economic inequalities, as plague became seen as a disease associated with poverty [23]. Plague remains a global health problem with the continuous enzootic transmission of *Y. pestis* and recurrent outbreaks in some places, as well as emerging antibiotic-resistant strains that reflect a deeply rooted disease ecological inheritance and health disparities. Future pandemic responses will need to focus on identifying and mitigating environmental and social pressures that foster the longevity of pandemics at both regional and global fronts.

## 4. New avenues for transdisciplinary research and data sources

Collaborative efforts between zoos, natural history museums, and biorepositories can contribute to understanding zoonotic disease host origins and transmission events [111]. Museum collections have provided valuable insight into the spread of antimicrobial resistance [112], chytrid fungus [113], Lyme disease [114], White Nose syndrome [115] and Sin Nombre virus [116]. In addition, they offer snapshots of biodiversity, spanning important time gaps between archaeological and modern outbreaks. Virtual communities such as Project ECHO's Museums and Emerging Pathogens in the Americas (MEPA) are transforming how collaborative research using biorepositories and specimen vouchers along with field collections can add value in predictive outbreak models [117].

## 5. Ethical considerations

While every field of research in this transdisciplinary One Health perspective has their own guidelines and standards for ethical research, we highlight two cross-cutting ethical concerns for marginalized and under-represented communities: (i) collaborative and inclusive research through community engagement and (ii) data sovereignty. Each of these ethical concerns are ongoing discussions [118–120]; however, we highlight some ways they have been implemented for a more inclusive and decolonized interpretation of the past.

Collaborative and inclusive research begins with community engagement. Consulting with descendant communities (broadly defined) can occur in all stages of research, from co-developing research agendas and identifying how the community will benefit to destructive sampling and data storage [120,121]. Co-interpretation of data, including authorship, can provide accurate cultural and behavioural contexts and avoid inappropriate language use. This has been highlighted within ‘omics’ research, where Indigenous communities are often relied on as a baseline of ‘non-Westernized’ versus ‘Westernized’ and are frequently described as ‘non-modern’, ‘non-industrialized’ or ‘traditional’ for comparative studies [122]. Such terminology relays to the broader audience that the community is somehow stagnant both culturally and genetically while progressing bias and inaccurate narratives [122]. The incorporation of ethnographic ethnohistoric records, oral traditions and languages in conjunction with archaeology and phylogeography have provided valuable insight where archaeological and phylogeographic evidence is lacking and have documented the longevity of zoonotic disease dynamics [123,124].

Data sovereignty is becoming increasingly important, especially for Indigenous communities [121]. Discussions of who has access to data, for how long and for what purpose at the onset of research can reduce conflict and harm. In addition, the researcher and community should discuss if future research can be pursued from the generated data as the misuse of data outside the scope of the project can have long-lasting and negative effects [119]. Indigenous consortiums and open-source platforms such as The Native BioData Consortium [125] and Mukurtu [126] are paving new paths for collaborative and ethical research. While these bioethics are becoming more prominent within archaeology, inclusive research needs to be further implemented in zoonotic outbreaks. This includes the sharing of zoonotic risk technologies and diagnostics with communities in higher-risk areas of spillovers to occur.

## 6. Conclusion

Conceptually and in practice, One Health breaks down barriers between human, animal and environmental health with the integration of traditionally divided disciplines, data sources and skillsets. As a field that already embraces the One Health triad in its broad questions and collaborative teamwork, as evident in the range of scientists who work together to reconstruct the past, archaeology is a natural fit for the One Health community. Bringing archaeology into One Health opens a discussion on how long-term ecological changes brought on by humans through niche construction can have lasting impacts and influence disease ecology. While this temporal dimension may not prevent novel zoonotic outbreaks, it does provide a deeper holistic foundation for disease ecology. Though some zoonotic pathogens may not be transmissible across multiple hosts due to host plasticity, zoonotic diseases that can infect ecologically and taxonomically diverse host range have proven to have devastating impacts. Now, with our accelerating population growth, rapid global travel, encroachment of wildlife habitats, antibiotic overuse and increasing socio-economic inequality, the biggest challenge for human health is preventing devastating overlapping pandemics. Foreseeing emerging events, therefore, cannot only begin with predicting likely pathogens to infect human populations, but rather must have a broader focus on pathogens that will probably infect secondary hosts that then transmit to humans, as well as pathogens that humans are likely to transmit to animals. Overlooking this multi-directional flow of microbes diminishes our understanding of spillover events.

This review has addressed how complex challenges in studying disease-scapes can be approached with long-term data. By incorporating palaeoecological, zooarchaeological and bioarchaeological tools, we can begin to untangle past human–animal–environment interactions and pathogen transmission over the past millennia. In so doing, the evolution of a disease-scape might then serve as a model in foreseeing future zoonotic events.

## Data Availability

This article has no additional data.
